# PCR-based method for targeting 16S-23S rRNA intergenic spacer regions among *Vibrio *species

**DOI:** 10.1186/1471-2180-10-90

**Published:** 2010-03-23

**Authors:** Maria Hoffmann, Eric W Brown, Peter CH Feng, Christine E Keys, Markus Fischer, Steven R Monday

**Affiliations:** 1 Division of Microbiology, Office of Regulatory Science Center for Food Safety and Applied Nutrition, US Food and Drug Administration, Paint Branch Parkway, College Park, MD 20740, USA; 2 Institute of Food Chemistry, University of Hamburg, Grindelallee 117, D-20146 Hamburg, Germany

## Abstract

**Background:**

The genus *Vibrio *is a diverse group of Gram-negative bacteria comprised of 74 species. Furthermore, the genus has and is expected to continue expanding with the addition of several new species annually. Consequently, it is of paramount importance to have a method which is able to reliably and efficiently differentiate the numerous *Vibrio *species.

**Results:**

In this study, a novel and rapid polymerase chain reaction (PCR)-based intergenic spacer (IGS)-typing system for vibrios was developed that is based on the well-known IGS regions located between the 16S and 23S rRNA genes on the bacterial chromosome. The system was optimized to resolve heteroduplex formation as well as to take advantage of capillary gel electrophoresis technology such that reproducible analyses could be achieved in a rapid manner. System validation was achieved through testing of 69 archetypal *Vibrio *strains, representing 48 *Vibrio *species, from which an 'IGS-type' profile database was generated. These data, presented here in several cluster analyses, demonstrated successful differentiation of the 69 type strains showing that this PCR-based fingerprinting method easily discriminates bacterial strains at the species level among *Vibrio*. Furthermore, testing 36 strains each of *V. parahaemolyticus *and *V. vulnificus*, important food borne pathogens, isolated from a variety of geographical locations with the IGS-typing method demonstrated distinct IGS-typing patterns indicative of subspecies divergence in both populations making this technique equally useful for intraspecies differentiation, as well.

**Conclusion:**

This rapid, reliable and efficient IGS-typing system, especially in combination with 16S rRNA gene sequencing, has the capacity to not only discern and identify vibrios at the species level but, in some cases, at the sub-species level, as well. This procedure is particularly well-suited for preliminary species identification and, lends itself nicely to epidemiological investigations providing information more quickly than other time-honoured methods traditionally used in these types of analyses.

## Background

*Vibrio *infections are becoming more and more common worldwide. The United States Centers for Disease Control and Prevention (CDC) estimates that 8,028 *Vibrio *infections and 57 deaths occur annually in the United States. Of these infections, 5,218 are foodborne in origin [1]. Three major syndromes, gastroenteritis, wound infection, and septicema, are caused by pathogenic vibrios. Within the genus *Vibrio*, *V. cholerae*, *V. parahaemolyticus *and *V. vulnificus *have long been established as important human pathogens in various parts of the world. Generally, these organisms are contracted after the patient has consumed raw or undercooked seafood, such as oysters, shrimp, and fish [2]. Hence, identification and subtyping of *Vibrio *isolates are of significant importance to public health and the safety of the human food supply.

In the last several years, an explosion of taxonomic studies have defined and redefined the members of the genus *Vibrio*. In 2004, Thompson et al. [2] introduced a classification strategy for vibrios that recommended, based on concatenated 16S rRNA gene sequencing, *recA*, and *rpoA *gene sequences, that the family *Vibrionaceae *be separated into four new families, *Vibrionaceae*, *Salinivibrionaceae*, *Photobacteriaceae *and *Enterovibrionaceae*. The new family *Vibrionaceae *is comprised solely of the genus *Vibrio*, which at that time consisted of 63 distinct species. To date, the genus *Vibrio *has expanded to include a total of 74 distinct species http://www.vibriobiology.net/ with several new *Vibrio *species being identified in the last four years [3-6]. As it likely that this trend will continue, it becomes increasingly important to have simple yet accurate identification systems capable of differentiating all *Vibrio *species.

An array of phenotypic and genomic techniques has become available for the identification of vibrios. Biochemical characteristics have been used to identify *Vibrio *species. However, *Vibrio *and other closely related species show similar phenotypic features and, subsequently, are not easily distinguished biochemically [7]. Studies in the past have shown that identification systems based on molecular genetic techniques, such as 16S rRNA gene sequencing, 16S-23S rRNA IGS regions, amplified fragment length polymorphism (AFLP) and multilocus sequence analyses (MLSA), are more discriminating than phenotypic methods and often provide more accurate taxonomic information about a particular strain [8-11]. Several investigators have used 16S rRNA gene sequences to study overall phylogenetic relationships of the *Vibrionaceae *[10,12,13]. However, within the genus *Vibrio*, many different species contain nearly identical 16S rRNA gene sequences rendering this method less reliable. Furthermore, as the number of known *Vibrio *species continues to rise, it becomes even more likely that sequence variation in the 16S rRNA gene will no longer be sufficient alone as a target for differentiation of closely related *Vibrio *species or subgroups within the same species [2]. Given the apparent short-comings of 16S rRNA gene sequence analyses for determining taxonomic and phylogenetic relationships of vibrios, an increasing premium is placed on the design, optimization, and deployment of subtyping schemes capable of more robust differentiation of vibrios. For bacteria with more than one rRNA operon, characterization of the 16S-23S rRNA IGS regions has been used successfully for subtyping closely related species. Due to variability in size and sequence of multiple IGS segments, size separation of PCR products spanning the IGS can enable effective differentiation of *Vibrio *species [14,15].

Previous studies using IGS fingerprinting have encountered several problems. Foremost is the formation of heteroduplex DNA artifacts (i.e., double-stranded DNA molecules comprised of individual strands arising from two separate PCR products that share significant homology such that annealing occurs) that make interpretation of results difficult and often intangible [16-19]. Furthermore, the earlier studies often relied on either agarose or polyacrylamide gel electrophoresis (PAGE) for resolution of amplicons, making the procedure a timely process, as well [20]. In this study, we present a novel PCR-based protocol that utilizes the IGS locus along with custom-designed, *Vibrio*-specific 16S and 23S rRNA gene PCR primers for the discrimination of *Vibrio *species. This improved system successfully eliminated the heteroduplexes frequently encountered in other IGS-typing protocols. Moreover, the system takes advantage of capillary gel electrophoresis technology for amplicon resolution in a more rapid and accurate manner than traditional gel electrophoresis-based approaches. An analysis of 69 *Vibrio *type strains demonstrated that the procedure reliably differentiates at the species level, allowing the generation of an IGS-type pattern database that could be used for subsequent identification of unknown *Vibrio *isolates. Finally, analysis of a collection of *V. parahaemolyticus *and *V. vulnificus *strains isolated from a variety of distinct geographical locales demonstrated intra-species IGS heterogeneity indicating that this protocol not only reliably differentiates at the species level but also at the subspecies level to some extent. Collectively, this report presents a *Vibrio *typing system that is versatile not only in identification of unknown isolates but also for epidemiological investigations, as well.

## Results

The study began by confirming that the 69 *Vibrio *type strains obtained from American Type Culture Collection (ATCC) and the Belgian Co-Ordinated Collection of Micro Organisms (BCCM) used in this study were correctly identified. The 16S rRNA gene sequence from each strain was successfully amplified and sequenced using eight additional sequencing primers. After contig assembly, BLAST (basic local alignment search tool) analysis of each product confirmed the actual identification of every type strain used in this study.

### Optimization and efficacy of the IGS-typing protocol

**Figure 1:**
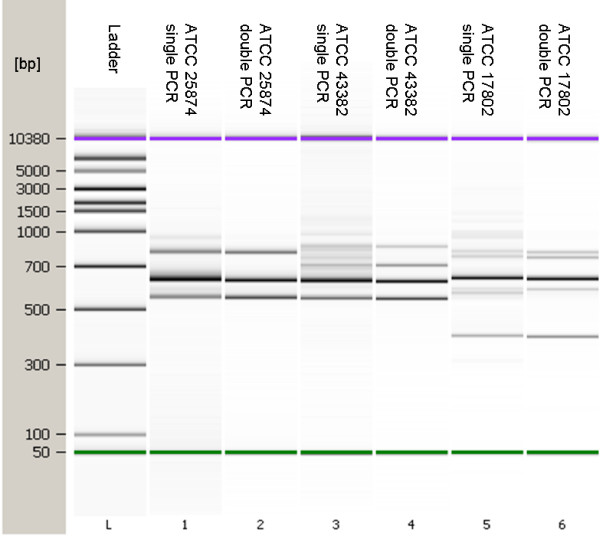
**This figure shows the successful elimination of heteroduplex artifacts following secondary PCR process**. Lanes one, three and five show IGS-pattern results following the initial PCR. Lanes two, four and six show IGS-type patterns for the same samples after completion of the one extra PCR amplification step. Lanes 1-2, *V. cholerae *ATCC 25874; lanes 3-4, *V. vulnificus *ATCC 43382; Lanes 5-6, *V. parahaemolyticus *ATCC 17802. Lane L, MW ladder.

**Figure 2:**
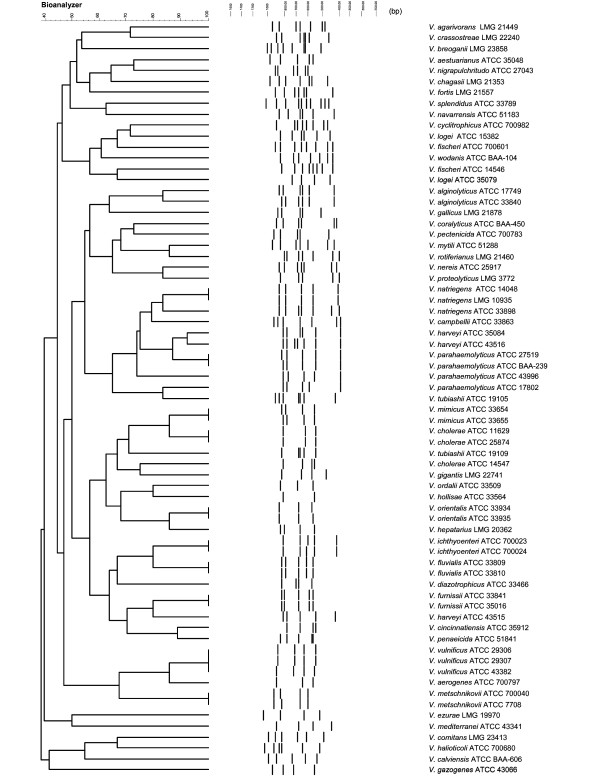
**BioNumerics-derived UPGMA Dendrogram generated from the results of the IGS-typing procedure using 69 *Vibrio *reference strains**. It is shown that all different species could be separated by virtue of their own unique 'specific-specific' IGS-type patterns. Parameters used to produce the dendrogram were: Dice (Opt:1.00%) (Tol 0.25-0.25%) (H>0.0% S>0.0%) [0.0%-100.0%].

Following identity confirmation, strains were subjected to the IGS-typing procedure designed in this study. Using the optimized PCR protocol, IGS amplicons were successfully generated from all *Vibrio *strains. These products were resolved using the Agilent BioAnalyzer 2100 capillary gel electrophoresis system. The system effectively separated the products, however, artifacts emerged that were not consistent with the products that should have been generated, as determined from nucleotide sequences available at the National Center for Biotechnology Information (NCBI) database. Presumably, these artifacts were a consequence of heteroduplex formation, a problem frequently associated with this type of analysis [16,19]. To circumvent this problem, a brief second-round amplification step was introduced that easily eliminated artifacts to produce crisp and resolute data patterns with the Agilent system (Figure 1). Analysis using BioNumerics yielded an unweight pair group method with arithmetic mean (UPGMA) dendrogram that demonstrated that the patterns generated were sufficiently different from one another so that all species could be separated by virtue of their own unique "species-specific" IGS-type patterns (Figure 2). Furthermore, these data buttress the notion that such a method focusing on the variable IGS regions of *Vibrio *species can be used to rapidly identify and distinguish individual species of important *Vibrio *pathogens.

**Figure 3:**
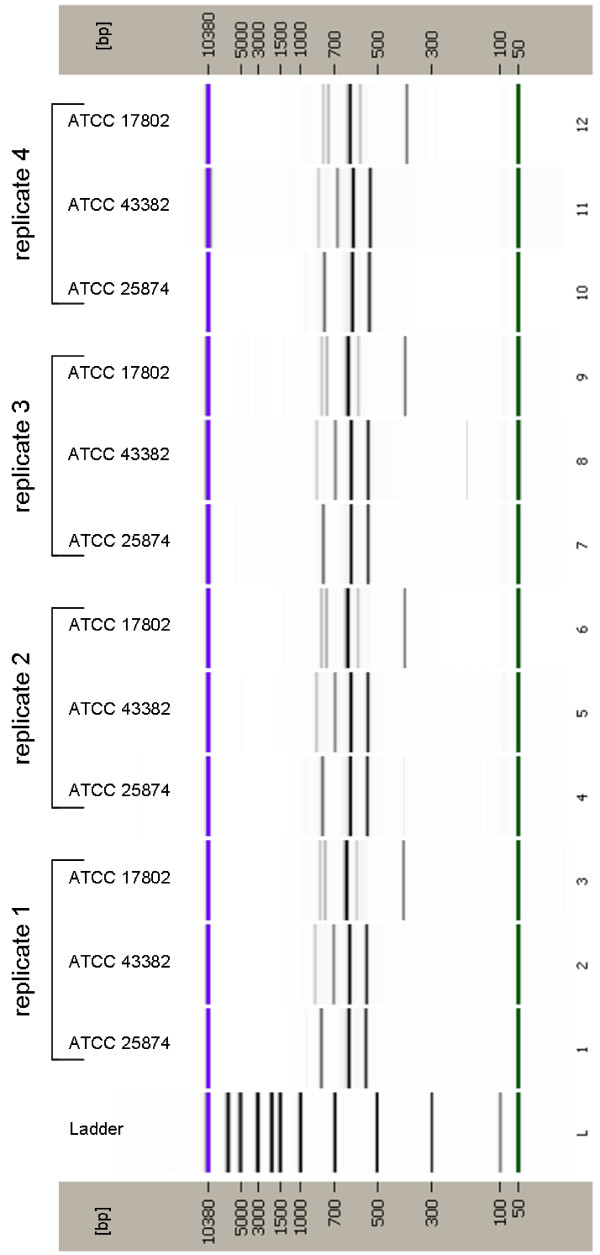
**Virtual gel picture of IGS-type patterns obtained from replicate analyses**. DNA was isolated from each strain four separate times and individually processed and evaluated for consistency in banding pattern. Lanes 1-3, replicate 1; Lanes 4-6, replicate 2; Lanes 7-9, replicate 3 and Lanes 10-12, replicate 4. Lanes 1, 4, 7 and 10: *V. cholerae *ATCC 25874; Lanes 2, 3, 8, and 11: *V*. *vulnificus *ATCC 43382; Lanes 3, 6, 9 and 12: *V. parahaemolyticus *ATCC 17802; Lane L, MW ladder.

Having demonstrated the efficiency of this method, the next step was to evaluate its fidelity. To this end, DNA was isolated from *V. cholerae *ATCC 25874, *V. vulnificus *ATCC 43382 and *V. parahaemolyticus *ATCC 17802 four separate times and individually processed (i.e., four individual biological replicates were produced). The cleaned PCR products from each of these replicates were analyzed simultaneously on the Bioanalyzer 2100. The resulting electropherograms and gel images generated by the Bioanalyzer 2100 revealed that all DNA templates derived from the same strain reproducibly yield the same IGS-type patterns (Figure 3). Furthermore, having found that these four species consistently yielded the same IGS-type patterns, the *Vibrio *type strains originally tested were subjected to an additional round of testing to assure that those patterns originally observed for the type strains were also consistently reproduced. As expected, the second round of testing yielded patterns identical to those originally observed. Clearly, based on these data, the method is both efficient and reliable.

### Differentiation of type strains by IGS-typing analysis

The 69 archetypal *Vibrio *strains used in this study represented 48 distinct species. In the course of evaluating these strains, it was noted in several cases that distinctly different IGS-patterns were obtained from the same species having homogenous 16S rRNA gene structure. For instance, *V. natriegens *ATCC 33898 differed by only a single base pair in 16S rRNA gene sequence structure from *V. natriegens *strains ATCC 14048 and LMG 10935 yet produced an IGS-pattern distinctly different than that observed for either ATCC 14048 or LMG 10935, both of which yielded identical IGS fingerprints (Figure 2). Similarly, *V. fischeri *strains ATCC 700601 and ATCC 14546 differed by only two base pairs in 16S rRNA gene structure but also demonstrated distinctly different IGS-patterns (Figure 2). However, these latter IGS-typic differences were not entirely unexpected, as several phenotypic differences between the isolates were also noted. For instance, ATCC 14546 demonstrated a phosphorescent phenotype on Photobacterium Agar (Sigma-Aldrich Laboratories, St. Louis, MO, USA) not noted by the ATCC 700601 strain. As with the *V. natriegens *and *V. fischeri *strains, *V. cholerae *strains ATCC 14541, ATCC 11629 and ATCC 25847 also shared identical 16S rRNA gene sequence homogeneity yet produced IGS-patterns that separated the strain ATCC 14541 away from the other two strains (ATCC 11629 and ATCC 25847). This might reflect the fact that ATCC 14541 was originally deposited with ATCC as *V. albensis *and later, erroneously, reclassified as *V. cholera *as a consequence of 16S rRNA gene sequence composition.

### Evidence of intra-species divergence by IGS-typing analysis

**Figure 4:**
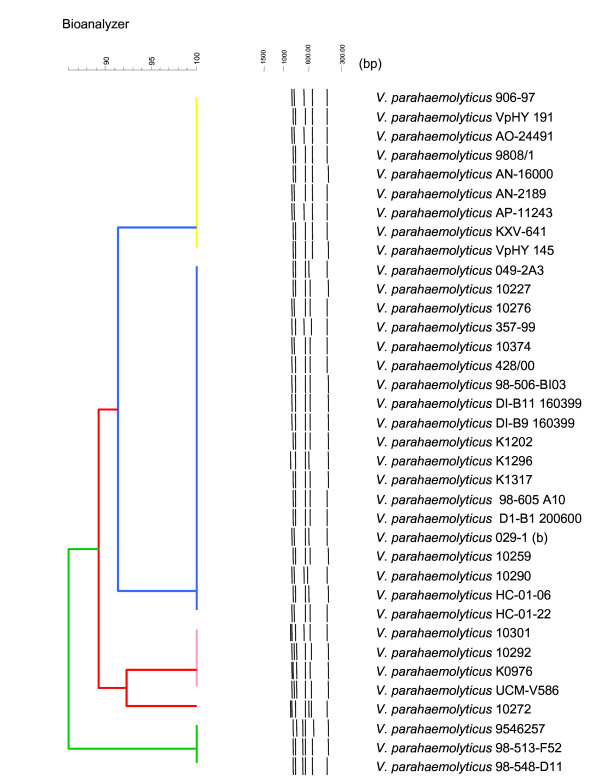
**BioNumerics-derived UPGMA dendrogram depicting results obtained from IGS-typing of the 36 *Vibrio parahaemolyticus *strains**. The UPGMA analysis separated the *V. parahaemolyticus *strains into five distinct clusters. Parameters used to produce the dendrogram were: Dice. (Opt:1.00%) (Tol 0.55%-0.55%) (H>0.0% S>0.0%) [0.0%-100.0%].

**Figure 5:**
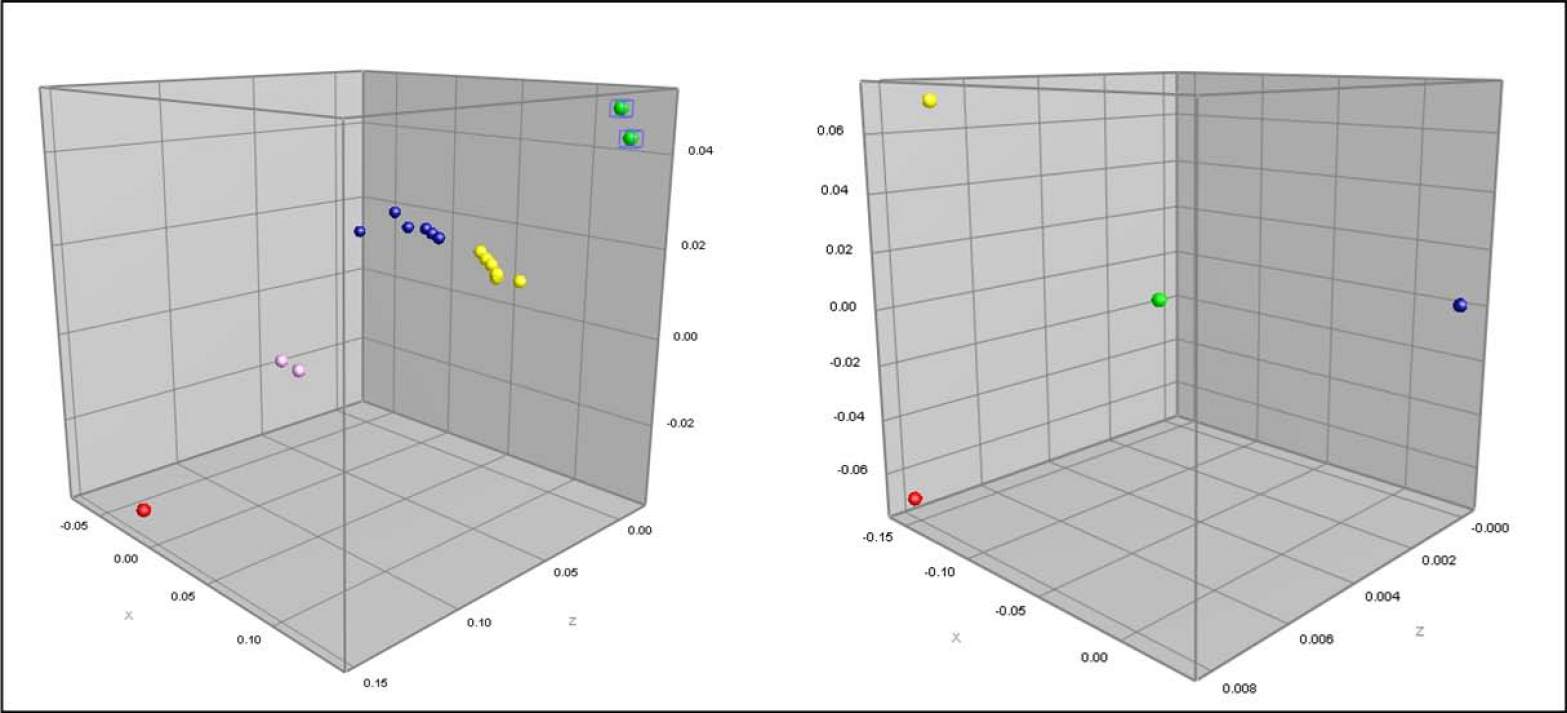
**BioNumerics-derived MDS representing results shown in UPGMA dendrogram of *V. parahaemolyticus *and *V. vulnificus***. The graphs shown of *V.parahaemolyticus *(Figure 4) and *V. vulnificus *(Figure 6) are depicted in a 3-dimensional format to better illustrate the genetic divergence between discrete clusters. *V. parahaemolyticus *is shown in the MDS on the left, while the MDS presented on the right is for *V. vulnificus*.

To further explore the extent of this intra-species divergence phenomenon, 36 strains of *V. parahaemolyticus *and *V. vulnificus*, obtained from various geographical locations, were evaluated by this IGS-typing method. Interestingly, a significant degree of heterogeneity in the IGS-pattern obtained from the *V. parahaemolyticus *isolates was observed, where the UPGMA analysis separated the *V. parahaemolyticus *strains into five distinct clusters (Figure 4). These clusters were more clearly observed in a 3D multidimensional scaling (MDS) analysis (Figure 5). In this view, distinct genetic partitions were noted, separated by substantial divergence among IGS-type patterns.

**Figure 6:**
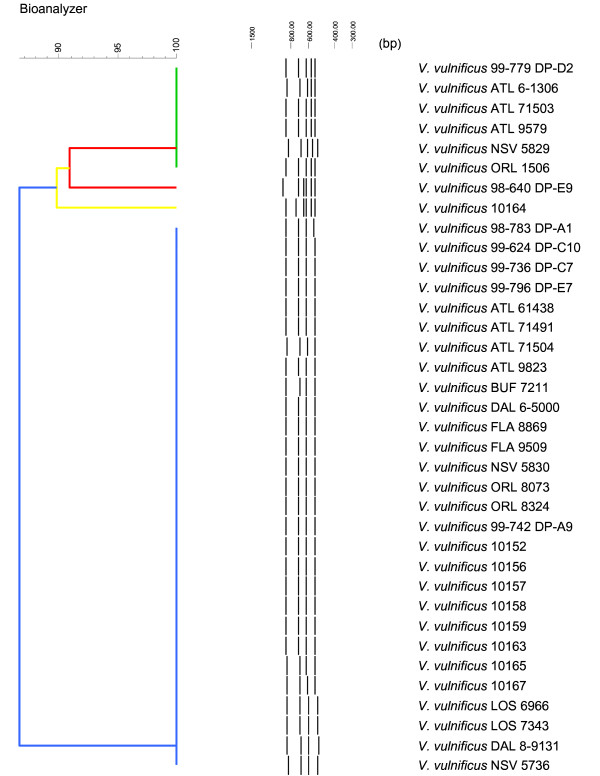
**BioNumerics-derived UPGMA dendrogram obtained following the IGS-typing of the 36 *V. vulnificus *strains**. The UPGMA analysis separated the *V. vulnificus *strains into four distinct clusters. Parameters used to produce the dendrogram were: Dice. (Opt:1.00%) (Tol 0.55%-0.55%) (H>0.0% S>0.0%) [0.0%-100.0%].

Similarly, although, to a lesser extent, the *V. vulnificus *strains demonstrated IGS-pattern heterogeneity that UPGMA analysis partitioned into four distinct clusters (Figure 5 and 6). Two of these four clusters were comprised of one strain, each signaling rare and unique genotypes for these patterns. Based on the limited population examined, it is notable that the four clusters can be easily distinguished since the IGS-types are substantially diverged and largely unique both in band composition and in major size shifts. A good example is pattern cluster one, which retains a band uniquely missing in pattern four (Figure 6).

## Discussion

The *Vibrio *genus is a complex group of marine-associated bacteria currently comprised of 74 species. The genus appears to be poised for continued growth as novel species are added regularly http://www.vibriobiology.net/. Consequently, this study was undertaken to develop a means by which these species could be efficiently, reliably, and accurately identified and differentiated. To date, analyses of IGS located between the 16S-23S rRNA gene loci have drawn considerable attention as one such means to accomplish this particular goal. Unfortunately, these analyses tend to be more laborious (i.e., restriction endonuclease analysis followed by probe-based detection) requiring a considerable time commitment. Moreover, many of these protocols generate extraneous artifacts that make interpretation of results often times difficult and unreliable.

**Figure 7:**
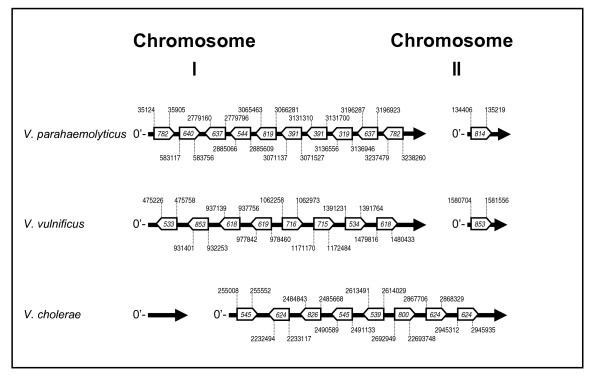
**Schematic depicting the relative size (bp), order, and chromosome position of 16S-23S rRNA IGS regions of 3 *Vibrio *species**. This figure shows the relevant genomic regions of *V. parahaemolyticus *RIMD 2210633 (Chromosome I: NC_004603; chromosome II: NC_004605), *V. cholerae *O395 (chromosome 1: NC_009456; chromosome 2: NC_009457) and *V. vulnificus *CMCP6 (chromosome 1: NC_004459; chromosome 2: NC_004460). Sequence coordinates denoting 16S-23S rRNA IGS primer binding sites are listed above and below their respective locus and correspond to the NCBI genome accessions provided here. IGS regions are denoted by open boxes with sizes (in bp) provided within. Directional orientation is indicated for both chromosomes by the 0 min start (0') to the left of each map.

To date, the most commonly used primers for the amplification of the IGS have been those described by Jensen et al. [21]. The 16S rRNA gene primer (G1) was generated for a highly conserved region of the 16S rRNA gene locus approximately 30-40 bp upstream of the IGS using the 16S rRNA gene sequence data generated by Dams et al [22] from a broad range of bacterial and eukaryotic genera (107 species). In contrast, as the 23S rRNA gene sequence is much less conserved than that of the 16S rRNA gene, the 23S primer (L1) was designed from the 23S rRNA gene sequences of only five bacterial and four plant species previously determined by Gutell et al [23]. As these primers were not based solely on *Vibrio *16S and 23S rRNA gene sequences, a new set of *Vibrio*-specific primers was designed from an alignment of 16S and 23S *Vibrio *rRNA gene sequences. PCR reactions were optimized using these primers such that the amplification products from four reference strains (*V. parahaemolyticus *BAA239 (O3:K6), *V. cholerae *ATCC 25874, *V. vulnificus *ATCC 43382 and *V. fischeri *ATCC 700601) were consistent with the number and sizes of those that could be theoretically derived from genomic sequences available at the NCBI database (*V. parahaemolyticus *RIMD 2210633 (Chromosome I: NC_004603; chromosome II: NC_004605), *V. cholerae *O395 (chromosome 1: NC_009456; chromosome 2: NC_009457), *V. vulnificus *CMCP6 (chromosome 1: NC_004459; chromosome 2: NC_004460) and *V. fischeri *ES 114 (chromosome 1: NC_006840; chromosome 2: NC_006841)). As an example, the chromosome coordinates, relative size, and number of IGS regions targeted by this assay for *V. parahaemolyticus*, *V. vulnificus*, and *V. cholerae *are depicted in Figure 7. In every case, IGS banding patterns correlated perfectly with expected fragment size (compare Figure 7 to Figures 1 and 3). Afterwards, the testing of each remaining reference species demonstrated unique banding patterns for all strains included. In the course of the optimization procedure, artifacts, the likely result of heteroduplex formation, were universally noted in the reaction products derived from these species. While several means by which heteroduplex formation could be eliminated or reduced are discussed in numerous publications [16,18,19], we found that only one [24], with some modification, produced results acceptable for use in this particular protocol. Subsequently, PCR products derived from the first amplification procedure were processed further with a second round of PCR optimized for heteroduplex elimination. Numerous testing of the two-round PCR procedure repeatedly yielded products devoid of transient artifacts, confirming that the process was suitable for and highly compatible with this type of analysis.

Previous IGS studies have relied on either agarose or PAGE for resolution of the amplicons generated by PCR-based IGS-typic analyses [14,25]. These methods can be somewhat cumbersome and require a lengthy amount of time to perform. To overcome this limitation, this protocol was engineered to take advantage of the rapid and sensitive capillary gel electrophoresis technology. Using the Agilent BioAnalyzer 2100 system, it was determined that a minimal amount of effort to more thoroughly clean the second round PCR products allowed this technology to deliver results that were at least as good as, if not better than, those obtained from traditional electrophoresis protocols. Furthermore, the Agilent system provided the additional benefit of a highly accurate and easily interpreted virtual gel-based result. That is, band interpretations were based on real genotypic differences defined by obvious deviations in band size, rather than subjective band 'bin' assignments so often incorporated with conventional agarose and PAGE.

While all reference species tested produced results that sufficiently differentiated them, as noted by the cluster analyses, we also determined, in a few cases, identical species having homogenous 16S rRNA gene sequence structure produced different IGS-type banding patterns. These patterns were often times substantially different such that identical species were separated widely on the resultant dendrograms. One explanation for this observation is that horizontal gene transfer (HGT) has occurred in the IGS of one isolate causing a deviation in its specific IGS-typic pattern. Additionally, as might be the case for the *V. fischeri *isolates that have identical 16S rRNA gene structure and, presumably, are the same species, when, in fact, they are not. In this particular case, one of the isolates displayed a phosphorescent phenotype while the other did not. Similarly, *V. cholerae *isolates demonstrated that all had identical 16S rRNA gene sequence structures yet only two of the isolates produced identical IGS-prints. The third *V. cholerae *ATCC 14541 produced a distinctly different banding pattern. Of particular interest is that this 'atypical' *V. cholerae *strain was originally deposited as *V. albensis*, not *V. cholerae*, underscoring the risks associated with speciating strains based solely on their 16S rRNA gene structure.

To explore intraspecies level IGS-typic divergence for several important *Vibrio *species, a comprehensive study characterizing the IGS-typic relationship within a population of 36 *V. parahaemolyticus *and 36 *V. vulnificus *isolates derived from different geographic locations was performed. As expected, these strains confirmed divergence of IGS-type patterns at the intraspecies level. Surprisingly, 15 different *V. parahaemolyticus *IGS-types, consisting of up to seven bands each, partitioned readily into five distinct clusters. This particular observation deviated significantly from an earlier study [26] proposing that *V. parahaemolyticus *was segregated into four clusters based solely on the four distinct IGS-patterns that were observed in their PAGE analysis. This significant difference in segregative ability allows a more powerful and discriminatory resolution of strains at the intraspecies level. Furthermore, the previous study [26], suggests that mismatches in the L1 (Jensen) primer [21] gave rise to a different and, presumably, an incorrect banding pattern from that generated when using their own primer set, although the L1/G1 pattern they present in their representation is clearly in agreement with the pattern that would be theoretically obtained from the NCBI genomic sequence of the *V. parahaemolyticus *RIMD 2210683 strain. Interestingly, the L1/G1 pattern presented in the earlier Jensen et al. study is entirely consistent with that of our own work, which is not entirely surprising as the sequence of L1 (and G1) are 100% complementary to the annealing sites of all 11 *V. parahaemolyticus *RIMD 2210683 rDNA loci.

We found in preliminary investigations of *V. vulnificus *that, although not to the degree of *V. parahaemolyticus*, the IGS-typing data also consisted of numerous (~10) unique patterns that partitioned nicely into four distinct clusters. Moreover, several of these isolates produced IGS-prints that consisted of five to six bands, significantly deviating from the pattern produced by our reference strains (*V. vulnificus *ATCC 43382, ATCC 29307 and ATCC 29306), as well as the theoretical pattern derived from the NCBI genomic sequence submission for *V. vulnificus *CMCP6 (NC_004459 and NC_004460), all of which consisted of a four band IGS-type pattern. These data may signal a reticulate evolutionary pattern for IGS sequences in this group of vibrios.

Notably, we found that the IGS-typing data derived from the *V. parahaemolyticus *study correlated nicely with the distributions of MLST sequence types (STs) previously generated for these strains, with no single ST observed in more than one cluster [27]. This finding was also noted in the *V. vulnificus *analysis [28]. For example, strains having ST16 converged into ribotype cluster one. Additionally in the case of *V. parahaemolyticus*, it is interesting to note that clusters two, three, four and five were primarily comprised of United States-derived isolates, indicating some degree of phylogeographic concordance with resultant IGS-prints (Figure 4). Taken together these observations suggest that it may, indeed, be possible to engage in epidemiological studies of outbreak strains using IGS-typing methodology. Furthermore, understanding and characterizing the relationship of these outbreak strains to their environmental counterparts might also be facilitated using this analytical strategy.

At present, it appears that, in complex genera consisting of numerous species, identification by monotypic analysis becomes increasingly more difficult and unreliable [2]. Clearly, this is the case for 16S rRNA gene sequence analysis of *Vibrio *strains, where unique and distinct species retain virtually identical 16S rRNA gene sequences, differing by as little as two to three (≥ 0.2%) base pairs. However, we have shown that it may be possible to discriminate at the species and intra-species levels using an analysis of IGS regions that is easy to perform, avoids cumbersome and time-consuming PAGE and agarose gel electrophoresis technologies and is devoid of the interfering artifacts that make accurate interpretation of results difficult at best. Moreover, this strategy incorporates a conservative analytical approach where only substantial, non-ambiguous results are considered in the interpretation of the analysis. In combination with a 16S rRNA gene sequencing analysis, the approach becomes even more powerful in the identification of species and, consequently, should prove invaluable for differentiation of species within a very complex *Vibrio *genus and for characterization of outbreak strains and isolates found in suspect environmental/food samples.

## Conclusion

This report describes a method that discriminates *Vibrio *species in a rapid and accurate manner. PCR amplification products derived from the 16S-23S rRNA genes IGS region could be analyzed using capillary gel electrophoresis technology to generate an IGS-typing pattern for each strain tested. The study showed that each of the species produced an IGS-typing pattern unique to itself that could be used to identify *Vibrio *species. Furthermore, the analysis could be performed more rapidly with the capillary gel electrophoresis procedure than either traditional agarose or PAGE protocols. The study also shows that there is sufficient intra-species IGS-typing pattern variation that differentiates at the subspecies, as well, especially when used in combination with 16S rRNA gene sequencing. As such, the procedure described in this report could be successfully used in preliminary epidemiological investigations, as well as other studies, to yield information more rapidly than other established subtyping methods requiring a considerably greater time commitment, such as pulsed field gel electrophoresis (PFGE), AFLP or MLSA.

## Methods

### Bacterial Strains, Growth Condition and Characterization

The 69 *Vibrio *type strains listed in Table 1 represented 48 species that served as reference taxa for this study. Isolates were obtained from ATCC and BCCM. Freeze-dried (lyophilized) cultures were revived according to protocols provided by the ATCC and BCCM curators. 16S rRNA gene sequencing (Amplicon Express, Pullman, WA, USA) was used as confirmation in assuring the identity of reference strains.

**Table 1:** ATCC and BCCM type strain collection used in this study

Designation	Strain*	Designation	Strain*
ATCC 700797	*V. aerogenes*	ATCC 33898	*V. natriegens*
ATCC 35048	*V. aestuarianus*	ATCC 14048	*V. natriegens*
ATCC 33840	*V. alginolyticus*	ATCC 51183	*V. navarrensis*
ATCC 17749	*V. alginolyticus*	ATCC 25917	*V. nereis*
ATCC BAA-606	*V. calviensis*	ATCC 27043	*V. nigrapulchritudo*
ATCC 33863	*V. campbellii*	ATCC 33509	*V. ordalii*
ATCC 11629	*V. cholerae*	ATCC 33934	*V. orientalis*
ATCC 25874	*V. cholerae*	ATCC 33935	*V. orientalis*
ATCC 14547	*V. cholerae*	ATCC 43996	*V. parahaemolyticus*
ATCC 35912	*V. cincinnatiensis*	ATCC 27519	*V. parahaemolyticus*
ATCC 700982	*V. cyclitrophicus*	ATCC 17802	*V. parahaemolyticus*
ATCC BAA-450	*V. coralyticus*	ATCC BAA-239	*V. parahaemolyticus*
ATCC 33466	*V. diazotrophicus*	ATCC 700783	*V. pectenicida*
ATCC 700601	*V. fischeri*	ATCC 51841	*V. penaeicida*
ATCC 14546	*V. fischeri*	ATCC 33789	*V. splendidus*
ATCC 33809	*V. fluvialis*	ATCC 19105	*V. tubiashii*
ATCC 33810	*V. fluvialis*	ATCC 19109	*V. tubiashii*
ATCC 35016	*V. furnissii*	ATCC 43382	*V. vulnificus*
ATCC 33841	*V. furnissii*	ATCC 29306	*V. vulnificus*
ATCC 43066	*V. gazogenes*	ATCC 29307	*V. vulnificus*
ATCC 700680	*V. halioticoli*	ATCC BAA-104	*V. wodansis*
ATCC 35084	*V. harveyi*	LMG 21449	*V. agarivorans*
ATCC 43515	*V. harveyi*	LMG 23858	*V. breoganii*
ATCC 43516	*V. harveyi*	LMG 21353	*V. chagasii*
ATCC 33564	*V. hollisae*	LMG 23413	*V. comitans*
ATCC 700023	*V. ichthyoenteri*	LMG 22240	*V. crassostreae*
ATCC 700024	*V. ichthyoenteri*	LMG 19970	*V. ezurae*
ATCC 15382	*V. logei*	LMG 21557	*V. fortis*
ATCC 35079	*V. logei*	LMG 21878	*V. gallicus*
ATCC 43341	*V. mediterranei*	LMG 22741	*V. gigantis*
ATCC 700040	*V. metschnikovii*	LMG 20362	*V. hepatarius*
ATCC 7708	*V. metschnikovii*	LMG 10935	*V. natriegens*
ATCC 33654	*V. mimicus*	LMG 3772	*V. proteolyticus*
ATCC 33655	*V. mimicus*	LMG 21460	*V. rotiferianus*
ATCC 51288	*V. mytili*		

*The following *Vibrio *type strains, used as reference strains in this study, were recently reclassified as different genera, as described here: *V. calviensis *became *Enterovibrio calviensis *[29]; *V. fisheri *became *Aliivibrio fisheri*, *V. logei *became *Aliivibrio logei*, *V. wodanis *became *Aliivibrio wodanis *[30]; and *V. hollisae *became *Grimontia hollisae *[31]. Through this paper, the former genus and species designations are used.

Thirty six *V. parahaemolyticus *and 36 *V. vulnificus *strains from various laboratories within the Food and Drug Administration (FDA) were also selected for this study. These strains, listed in Table 2, were very well characterized at the FDA (Dauphin Island AL) [20,27]. The strains were grown overnight with shaking (112 rpm) in Luria Bertani (LB; DIFCO Laboratories) medium at 37°C. Thiosulfate-Citrate-Bile Salts-Sucrose (TCBS; DIFCO Laboratories) Agar was used also as a selective agar to differentiate *V. vulnificus *and *V. parahaemolyticus *strains. Further confirmation of strain identity based on biochemical identification was performed using the standardized API 20 E identification system (bioMérieux, L'Etoile, France) and the Pathotec^R ^Cytochrome Oxidase Test (Remel, Lenexa, KS, USA) using pure cultures of isolated colonies grown on LB for 16-20 hours at 37°C according to the protocol provided by suppliers. API 20E identification was performed using the Apiweb™ identification software.

**Table 2:** *V. parahaemolyticus *and *V. vulnificus *strains used in this study

*V. parahaemolyticus *strains				*V. vulnificus *strains			
Strain	Country*	Source	**ST**^#^	Strain	Country*	Source	**ST**^#^
AN-16000	Bangladesh	Clinical	3	98-783 DP-A1	USA-LA	Environ.	26
AN-2189	Bangladesh	Clinical	3	99-742 DP-A9	USA-MS	Environ.	22
AO-24491	Bangladesh	Clinical	3	99-736 DP-C7	USA-FL	Environ.	34
AP-11243	Bangladesh	Clinical	51	99-624 DP-C10	USA-TX	Environ.	17
428/00	Spain	Clinical	17	99-779 DP-D2	USA-LA	Environ.	51
UCM-V586	Spain	Environ.	45	99-796 DP-E7	USA-FL	Environ.	22
9808/1	Spain	Clinical	3	98-640 DP-E9	USA-LA	Environ.	24
906-97	Peru	Clinical	3	ATL 6-1306	USA-FL	Clinical	16
357-99	Peru	Clinical	19	ATL 71503	USA-FL	Clinical	16
VpHY191	Thailand	Clinical	3	ATL 9579	USA-TX	Clinical	19
VpHY145	Thailand	Clinical	3	ATL 61438	USA-TX	Clinical	N/A
KXV-641	Japan	Clinical	3	ATL 9823	USA-LA	Clinical	37
98-605-A10	USA-CT	Environ.	31	ATL 71491	USA-TX	Clinical	32
9546257	USA-CA	Clinical	32	ATL 71504	USA-LA	Clinical	32
049-2A3	USA-OR	Environ.	57	BUF 7211	USA-FL	Clinical	N/A
98-506-B103	USA-VA	Environ.	30	DAL 8-9131	USA-TX	Clinical	N/A
98-548-D11	USA-MA	Environ.	34	DAL 6-5000	USA-LA	Clinical	18
98-513-F52	USA-LA	Environ.	34	FLA 8869	USATX	Clinical	40
DI-B9 160399	USA-AL	Environ.	25	FLA 9509	USA-LA	Clinical	40
DI-B11 160399	USA-AL	Environ.	54	LOS 6966	USA-TX	Clinical	2
DI-B-1 200600	USA-AL	Environ.	23	LOS 7343	USA-LA	Clinical	32
HC-01-22	USA-WA	Environ.	43	NSV 5736	USA-AL	Clinical	33
HC-01-06	USA-WA	Environ.	41	NSV 5830	USA-FL	Clinical	52
K0976	USA-AK	Environ.	4	NSV 5829	USA-FL	Clinical	16
K1202	USA-AK	Environ.	43	ORL 1506	USA-FL	Clinical	16
K1296	USA-AK	Environ.	9	ORL 8324	USA-FL	Clinical	37
K1317	USA-AK	Environ.	5	ORL 8073	USA-FL	Clinical	N/A
029-1 (b)	USA-OR	Environ.	36	10152	USA-WA	Clinical	N/A
10290	USA-WA	Clinical	37	10156	USA-WA	Clinical	N/A
10292	USA-WA	Clinical	50	10157	USA-WA	Environ.	N/A
10227	USA-WA	Environ.	N/A	10158	USA-WA	Environ.	N/A
10259	USA-WA	Clinical	N/A	10159	USA-WA	Clinical	N/A
10272	USA-WA	Environ.	N/A	10163	USA-WA	Environ.	N/A
10276	USA-WA	Environ.	N/A	10164	USA-WA	Clinical	N/A
10301	USA-WA	Environ.	N/A	10165	USA-WA	Clinical	N/A
10374	USA-WA	Clinical	N/A	10167	USA-WA	Clinical	N/A

*USA:United States; AL: Alabama; AK: Alaska; CA: California; CT: Connecticut; FL: Florida; LA: Louisanna; MA: Maryland; MS: Mississippi; OR: Organ; TX: Texas; VA: Virginia; WA: Washington ^#^ST: sequence type

Genomic DNA was isolated from all strains using the ZR Fungal/Bacterial DNA kit (Zymo Research, Orange, CA) according to the manufacturer's protocol. Purified DNA was quantified spectrophotometrically using a Nano Drop-1000 Spectrophotometer (NanoDrop Technologies, Inc., Wilmington, DE, USA) and diluted to a final concentration of 100 ng/μl using DNase/RNase-free double-distilled water (ddH_2_O).

### 16 S rRNA gene sequencing

Oligonucleotide primers for amplification of the 16S rRNA gene and subsequent sequencing were designed using conserved sequences detected within a Clustal X nucleotide alignment of the *Vibrio *16S nucleotide sequences obtained from the NCBI database. 16S rRNA gene sequences from 15 separate *Vibrio *species were used for the sequence alignment. Derived primer sequences were evaluated for predicted efficiency using the NetPrimer computer software (Premier Biosoft International, Palo Alto, CA, USA). The primers used for PCR amplification were: 16SF [5'-GTTTGATCATGGCTCAGATTG-3'] and 16SR [5'-CTACCTTGTTACGACTTCACC-3'].

The PCR was performed in a 50 μl volume with HotStarTaq Master Mix (Qiagen, Valencia, CA, USA) containing 400 μM dNTP (each of dATP, dCTP, dGTP and dTTP), 5 U of HotStart *Taq *Polymerase (Qiagen), 1x *Taq *polymerase buffer (Qiagen), 2.5 mM MgCl_2 _and a 300 nM concentration of each primer with ~100 ng of DNA template. The optimized amplification program began with a 95°C for 15 min enzyme activation step. To minimize PCR products derived from mispriming events, the actual amplification was initiated with a 'touchdown' PCR step consisting of 10 cycles at 95°C for 30 second (sec), 72°C-63°C (decreasing 1°C/cycle) for 20 sec and 72°C for 1.00 min followed by 35 cycles of 95°C for 30 sec, 63°C for 20 sec and 72°C for 1.00 min. The process was finished with a single cycle at 72°C for 2 min and stored at 4°C until analyzed.

Both strands of amplified PCR products were sequenced by Amplicon Express (Pullman, WA, USA) using Big Dye chemistry with 4 forward and 4 reverse target-specific sequencing primers (Table 3) in an ABI 3730 XL DNA sequencer according to the manufacturer's directions. DNA sequences were edited and assembled using DNAStar, Inc. (Madison, WI) Lasergene SeqMan II 5.07 sequence analyses software. After analyzing and assembling the respective sequences, a consensus sequence was used to query the NCBI BLAST database at NCBI to reconfirm reference strain identity.

**Table 3:** 16S rRNA gene sequencing primers used in this study

16SR1	5'-CAATATTCCCYACTGCTGC-3'
16SR2	5'-CATCGTTTACGYCGTGGACT-3'
16SR3	5'-GCTCGTTGCGGGACTTA-3'
16SR4	5'-GCTACCTTGTTACGACTTCACC-3'
16SF1	5'-GCRGGCCTAAYACATGCA-3'
16SF2	5'-TGAGACACGGYCCAGACTCCTAC-3'
16SF3	5'-GTAGCGGTGAAATGCGTAGA-3'
16SF4	5'-TGTCGTCAGCTCGTGTYGTG-3'

### IGS-typing PCR

IGS PCR primers were designed using conserved sequences detected within a Clustal X nucleotide alignment of both the *Vibrio *16S rRNA gene and 23S rRNA gene nucleotide sequences obtained from NCBI. The 16S rRNA gene and 23S rRNA gene sequences from 15 separate *Vibrio *species (i.e., *V. navarrensis*, *V. vulnificus*, *V. fischeri*, *V. logei*, *V. mediterranei*, *V. pelagius*, *V. splendidus*, *V. lentus*, *V. harveyii*, *V. parahaemolyticus*, *V. natriegens*, *V. ordalii*, *V. hollisae*, *V. fluvialis *and *V. cholerae*) and *E. coli *were used for the sequence alignment. Derived primer sequences 16S.6 [5'-ACTGGGGTGAAGTCGTAACA-3'] and 23S.1 [5'-CTTCATCGCCTCTGACTGC-3'] were evaluated for predicted efficiency using the NetPrimer Computer software.

PCR was performed in a 50 μl volume containing 300 μM dNTP, 5 U of HotStart *Taq *Polymerase, 1 × *Taq *polymerase buffer, 1.5 mM MgCl_2 _and a 300 nM concentration of each primer with ~100 ng of DNA template. The amplification program was 95°C for 15 min, 10 cycles at 95°C for 30 sec., 73°C-64°C (decreasing 1°C/cycle) for 10 sec and 72°C for 45 sec. Afterwards, complete amplification was achieved with 34 cycles of 95°C for 30 sec, 64°C for 10 sec and 72°C for 45 sec. The process was finished with a single cycle at 72°C for 1 min and stored at 4°C.

Heteroduplex formation was resolved with an additional amplification cycle [24] where the initial PCR products were diluted 1:5 in a 30 μl volume and subjected to a single amplification cycle of 95°C for 15.00 min, 64°C for 1.00 min and 72°C for 10.00 min in a similar reaction mixture containing 600 nM primer concentration. Afterwards, the PCR products were purified using QIAquick PCR Purification Kit (Qiagen) according to the manufacturer's protocol and eluted into 10 μL of nuclease-free Water.

### Analysis of IGS-typing fingerprints

IGS PCR amplicons were resolved by capillary gel electrophoresis using the Agilent BioAnalyzer 2100 and the Agilent DNA 7500 Assay Protocol (Agilent Technologies, Inc., Santa Clara, CA, USA). Using the BioAnalyzer 2100 integrated computer software, electropherograms and gel patterns were generated depicting the resulting PCR products derived from the IGS-typing reaction. Faint bands comprising less than 5% of the total DNA concentration and measuring less than 1 ng/ul were discarded prior to performing the analysis using BioNumerics fingerprinting software 5.10 (Applied Mathematics, Sint Martens Latem, Belgium). BioAnalyzer data was imported directly into the BioNumerics software using a custom script written by Applied Math for the BioNumerics software. The BioNumerics software used the Dice similarity coefficient to generate the UPGMA dendrograms presented in this study with Dice parameters: Optimization (Opt): 1.00%, Tolerance (Tol). 0.25% - 0.25% for the reference strains, and Opt: 1.00%, Tol. 0.55% - 0.55% for the 36 *V. vulnificus *and 36 *V. parahaemolyticus *strains.

## List of abbreviations used

AFLP: amplified fragment length polymorphism; ATCC: American Type Culture Collection; BCCM: Belgian Co-Ordinated Collection of Micro-Organisms; BLAST: basic local alignment search tool; CDC: The United States Centers for Disease Control and Prevention; DNA: deoxyribonucleic acid; FDA: Food and Drug Administration; HGT: horizontal gene transfer; IGS: intergenic spacer; LB: Luria Bertani; LMG: Laboratory of Microbiology Gent Bacteria Collection; MDS: 3D multidimensional scaling; MLSA: multilocus sequence analyses; NCBI: National Center for Biotechnology Information; Opt: Optimization; PAGE: polyacylamide gel electrophoresis; PCR: polymerase chain reaction; *recA*: recombinase A; *rpoA*: DNA-directed RNA polymerase subunit alpha; rRNA: ribosomal ribonucleic acid; ST: sequence type; S: Svedberg; TCBS: Thiosulfate-Citrate-Bile Salts-Sucrose; Tol: tolerance; UPGMA: unweight pair group method with arithmetic mean; *V*.: *Vibrio*.

## Authors' contributions

All authors played an integral part of project conception and method development described in the article. Each author has read and approved the final version of the manuscript. Specifically, MH performed the experimental procedures of the method development, including subsequent validation, and optimization, as well as the data analysis and interpretation of the results, and preparation of the manuscript. PCHF assisted with the microbiology component of the study and provided editorial assistance with the manuscript. CEK assisted with the data analysis and figure compilation. Following consultation with the authors, SRM, EWB and MF designed the experimental procedures for the study, participated in the data analyses and interpretation. SRM assisted with the method development and preparation of the manuscript.
